# Feasibility of Implementing a Dietary Intervention in Older Adults with Mild Depressive Symptoms and Low Activity: Lessons Learned

**DOI:** 10.3390/nu18213694

**Published:** 2026-11-07

**Authors:** Courtney L. Millar, Alyssa B. Dufour, Alex Wolfe, Jasmin Kuo, Douglas P. Kiel, Lewis A. Lipsitz

**Affiliations:** 1 Department of Medicine, Beth Israel Deaconess Medical Center, Harvard Medical School, Boston, MA 02215, USA; alyssadufour@hsl.harvard.edu (A.B.D.); kiel@hsl.harvard.edu (D.P.K.); lipsitz@hsl.harvard.edu (L.A.L.); 2 Hinda and Arthur Marcus Institute for Aging Research, Hebrew SeniorLife, Boston, MA 02131, USA; alexwolfe@hsl.harvard.edu; 3 School of Medicine, Tufts University, Boston, MA 02111, USA; kuojasmin@gmail.com

**Keywords:** feasibility, aging, depression, dietary intervention, trials

## Abstract

**Background/Objectives**: Several dietary interventions of blueberry intake have been performed for a wide variety of clinical outcomes (e.g., cardiovascular, bone), but none have been conducted in older adults with mild depressive symptoms and low activity. Such individuals may represent a unique group who may be in particular need of a lifestyle change, but lack the motivation to engage in an intervention. Thus, our goal was to test the feasibility of a dietary intervention in which older adults with mild depressive symptoms and low activity were randomly assigned to consume 48 g of freeze-dried blueberry or placebo powder each day for 3 months. **Methods**: Individuals were recruited by various methods (e.g., targeted web-based advertisements, flyers, etc.). Our primary assessment of feasibility was retention rate (e.g., the percentage of individuals that completed the intervention out of the total randomized); however, other metrics like recruitment rate, safety, and compliance were also evaluated. We pre-specified that the study would be considered feasible if 80% of the individuals that were randomized to the intervention completed the study. **Results**: Our study found that 76% (95%CI: 59–93; n = 22 out of 29) of the individuals randomized to the intervention went on to complete the intervention, which is close to, but does not satisfy our pre-specified feasibility threshold. Among individuals that completed the study, compliance with the study powder over the 3 months was relatively high (≥87% in both groups). There were no serious adverse events, and only 10 adverse events that occurred similarly across groups (n = 6 in placebo, n = 4 in blueberry). **Conclusions**: Together, our data provides evidence that a dietary intervention supplementing a relatively high dose of blueberry powder in a unique population of older adults is possible and adherence was high among those that completed the intervention.

## 1. Introduction

Aging is a universal phenomenon that is associated with the development of serious and important geriatric conditions. Although pharmacological therapeutics (e.g., anti-hypertensive, anti-depressants) have been identified to treat aging conditions (e.g., cardiovascular diseases, depression, respectively), use of pharmaceuticals in older adults comes with challenges, like polypharmacy, side effects, and/or contraindications [1]. Since older adults appear to prefer lifestyle strategies over pharmaceuticals [2] to help improve geriatric outcomes like depression [3], identification of alternative lifestyle strategies as therapeutic avenues for diseases of aging are needed to avoid challenges commonly associated with pharmaceuticals.

Dietary supplementation is a relatively safe and convenient alternative strategy that may be useful in older adults with depressive symptomology. While some dietary interventions for depressive symptoms have been tested in older adults as reviewed by Li et al. [4], the literature would benefit from confirming the feasibility of delivering a dietary intervention in such a population, one which may come with unique challenges such as retention and compliance. Trying to recruit enough older adults into a clinical trial is challenging [5]. Recruiting older adults with specific health conditions (i.e., mild depressive symptoms) imposes further recruitment difficulties by limiting the potential pool of older adults that may be eligible for the study. Furthermore, those with mild depressive symptoms experience anhedonia and lack of motivation [6,7,8]. Such individuals may be difficult to recruit and retain in a clinical intervention.

In addition to recruitment challenges that can arise with a population of older adults with depressive symptoms, recruiting older adults for dietary interventions is also extremely difficult. Older adults often develop set patterns for their dietary preferences, and they may be resistant to change [9]. Additionally, supplementation with one “food” as an intervention (e.g., daily blueberry intake) over a long-term period can be monotonous and participants may grow weary of these foods. Thus, compliance with a dietary intervention may be poor, particularly for older adults.

In light of these challenges, it is necessary to understand the feasibility (e.g., retention rates, recruitment challenges, compliance, and safety) of implementing a dietary interventions in older adults with geriatric conditions in order to confirm if such interventions are practical, therapeutic avenues. Therefore, we performed a pilot study in older adults with mild depressive symptoms and low activity to help determine the feasibility of a dietary intervention of blueberry intake in a population who may be difficult to recruit, have poor adherence, and grow weary of a monotonous food intervention. Blueberries were chosen as our dietary intervention since they are a relatively high source of dietary fiber and flavonoid antioxidants, which have been associated with reduced depression severity [10,11,12,13,14,15,16]. We hypothesized that at least 80% of the participants enrolled would complete the 3-month intervention, and that we would evaluate compliance, safety, and method of intake to further describe feasibility.

## 2. Materials and Methods

### 2.1. Study Design

The Berries and Steps Study was a 3-month, double-blind, randomized, parallel-arm, pilot study recruiting older adults with mild depressive symptoms and low activity. Participants were randomly assigned to consume either 48 g of freeze-dried blueberry powder or placebo powder each day for 3 months. The study was registered at Clinicaltrials.gov (NCT05735587; registered on 30 January 2023) and approved by the Advarra IRB (#Pro00064749; initial approval on 28 July 2022). All participants provided informed consent.

### 2.2. Recruitment and Eligibility

Individuals were primarily recruited in the Greater Boston area, through an online recruitment firm (i.e., BuildClinical) that uses internet browsing history to deliver targeted advertisements. Participants were also recruited through flyers and presentations at senior housing sites, participant registries, and various other online advertisements. Older adults (age ≥ 65 years) were eligible if they reported lack of motivation to exercise, sat for a majority of the day (≥8 h), and displayed mild depressive symptoms (defined as the scores of 4–16 points on either the Center for Epidemiological Depression Scale, CES-D; or the CESD-Revised questionnaires). The range of 4–16 points was used to identify individuals that do not have major depression, which is typically identified as a score ≥ 16 points [17,18,19], but still have room to improve on the scale without having a floor effect. Both scales were used because the CES-D is most cited compared to the CESD-revised, but the CESD-revised scale aligns more closely with the Diagnostic and Statistical Manual of Mental Disorders updated guidelines. Thus, inclusion of both scales allow for data comparisons across the larger literature and most closely identify individuals with mild depressive symptoms.

Participants were excluded from the study if they had a median daily step count > 7500 steps/day (as measured by the ActiGraph). There were 2 individuals that were on the cusp of the cut off for the step count eligibility criteria. The step count measure is evaluated by an electronic device, which is subject to error. Thus, it is possible that the participant did in fact meet our eligibility criteria and after consultation with our IRB, these individuals were included. Participants were also excluded if they had cognitive impairment (defined as Montreal Cognitive Assessment [20], MoCA < 22 points), self-reported history of inflammatory bowel disease/syndrome, major depression, bipolar, schizophrenia, or other psychotic disorders, self-reported diabetes, allergy to intervention or control products, recent use (within the last 3 months) of antibiotics or probiotics, current substance use disorder (Drug Abuse Screening Test [21,22], DAST-10 > 2 points), current alcohol use disorder (Alcohol Use Disorders Identification Test—Consumption [23,24], AUDIT-C ≥ 4 points), unstable anti-depressant use (e.g., change in medication within last 3–6 months), current homicidal or suicidal ideation (assessed via the P4 Suicidality Screener [25]), current psychosis (via the Psychosis and Hallucinations Questionnaire [26], PHQ > 12 points), or manic symptoms (assessed by the Mood Disorder Questionnaire [27], MDQ > 5 points).

### 2.3. General Study Procedure

Participants were asked to complete an initial telephone screener and an in-person screener, that included a baseline assessment of daily step count via a digital watch over approximately a 2-week period. Each individual’s median step count over the ~2 week period was estimated and used as an eligibility criteria. If eligible, participants completed a baseline assessment that included anthropometric measures, symptoms, and various self-report questionnaire assessments (e.g., motivation level via Motives for Physical Activities Measure—Revised, social connectedness via Multidimensional Scale of Perceived Social Support and cognitive status via MoCA). Additionally, a fasting blood draw was performed. Participants were randomized to consume 48 g of a randomly assigned powder (blueberry or placebo) each day for the next 3 months, while wearing a digital watch. Re-assessment of anthropometrics, symptoms, and questionnaires were performed at mid-point (~6 weeks) and final follow-up (~3 months), as well as a fasted blood draw. Also, there were telephone check-ins at 1 and 2 months. A more thorough description of the protocol is described elsewhere [28].

### 2.4. Study Powders

The blueberry and placebo powders were provided by the United States Highbush Blueberry Council. However, they did not have any role in the design or analysis of this study. The blueberry powder consisted of whole blueberries, that were freeze-dried and ground into a powder. The placebo powder was a matched powder that primarily consisted of maltodextrin, sugar, coloring, and flavoring. Powders were similarly packaged (24 g/packet), iso-caloric, and tasted and looked similar. Participants were asked to consume 2 packets of powder (~48 g) all at once in the morning, if possible. To avoid boredom and promote compliance, they were allowed to consume their respective powder through various methods throughout the intervention. They were given example recipes of how to consume the powder as previously described [28].

### 2.5. Blinding and Randomization

All study participants were randomly assigned to consume either the placebo powder or the blueberry powder. Randomization was done in blocks of 4 in an attempt to control for time/season. The randomization scheme was generated in SAS 9.4. Both powders were packaged in the same foil packets that only differed by a letter. One letter denoted the placebo powder packets and another letter denoted the blueberry powder packets. The code that distinguished the two powders was maintained by the US High Bush Blueberry Council until all analyses were completed. Therefore, all participants, study staff, outcome assessors, and analysts were blinded.

### 2.6. Feasibility Outcomes

Feasibility of our intervention was evaluated through recruitment/retention rates, compliance estimates, and safety measures that were tracked throughout the entire study period. Retention rate was selected as the primary feasibility outcome.

Retention, Recruitment, and Drop-out Rates: The retention (% of randomized individuals that completed the intervention) and recruitment rates (% of screened individuals that were enrolled in the study) were estimated at various stages of our enrollment period: telephone screening, in-person screening, and randomization. The number of individuals that dropped out of the intervention after randomization was also tabulated. Our primary aspect of feasibility was retention rate after randomization, which was defined as number of participants that completed the intervention out of the total randomized.

Compliance and Method of Powder Consumption: Participant compliance with the study powder was primarily assessed by self-reported compliance logs. Participants were instructed to complete the compliance log each day, recording if the powder was taken that day, how much powder was taken on a given day (%), and the method the powder was used (water, almond milk, cereal, smoothie, jam, muffin or other). Our primary analysis of compliance was performed on the individuals that completed the intervention. Because this may have biased our results, we included an analysis of compliance in all randomized individuals for the time that they were on the study, providing data was available. When complete data on compliance was not available (n = 4 of 7 individuals), we conservatively assumed that participants only took one dose of the study powder, which was confirmed via follow-up telephone call. A compliance percentage was estimated by dividing the number of doses consumed by the number of intended doses. If participants reported that they only consumed 50% of the prescribed dose, it was considered only half of the intended dose was consumed. To further describe how the powder was consumed, we also evaluate the percentage of time the powder was consumed by each method of powder consumption (e.g., water, almond milk, cereal, smoothie, jam, muffin or other).

Compliance with Wearable Device: Participants were instructed to wear the Actigraph GT9X Link device each day while on the study and wear-time data was downloaded from the devices at each in-person visit. For individuals that completed the study, compliance percentage with the wearable device was estimated by dividing the number of days the participant wore the device by the number of days the participant was on the intervention. Although participants wore the watch during the entire study including the run-in period, compliance percentage with the wearable device only evaluates wear-time between baseline and follow-up of the intervention period.

Safety: To evaluate safety, all adverse events and serious adverse events (AE, SAE, respectively) were tracked during the study. A detailed description of how AEs were defined and identified is provided elsewhere [28]. The number and percentage of AEs or SAEs were tabulated overall and by group.

### 2.7. Statistical Analysis and Basis of Sample Size

For all of those enrolled in our study, participant characteristics are reported as mean ± standard deviation or as n (%), as appropriate. The feasibility outcomes with 95% confidence intervals were tabulated. This is a descriptive study, so no formal quantitative analysis was performed. We pre-specified that if the retention rate of those randomized was ≥80%, the intervention would be considered feasible. Our sample size was chosen to provide a practical basis for developing variance measures of the planned outcomes. Such data are needed to design a subsequent larger trial aiming to establish efficacy of our intervention. Since our study is a preliminary pilot study, no formal power analysis was performed, which is further described elsewhere [28].

## 3. Results

### 3.1. Study Participant Flow Chart

**Figure 1:**
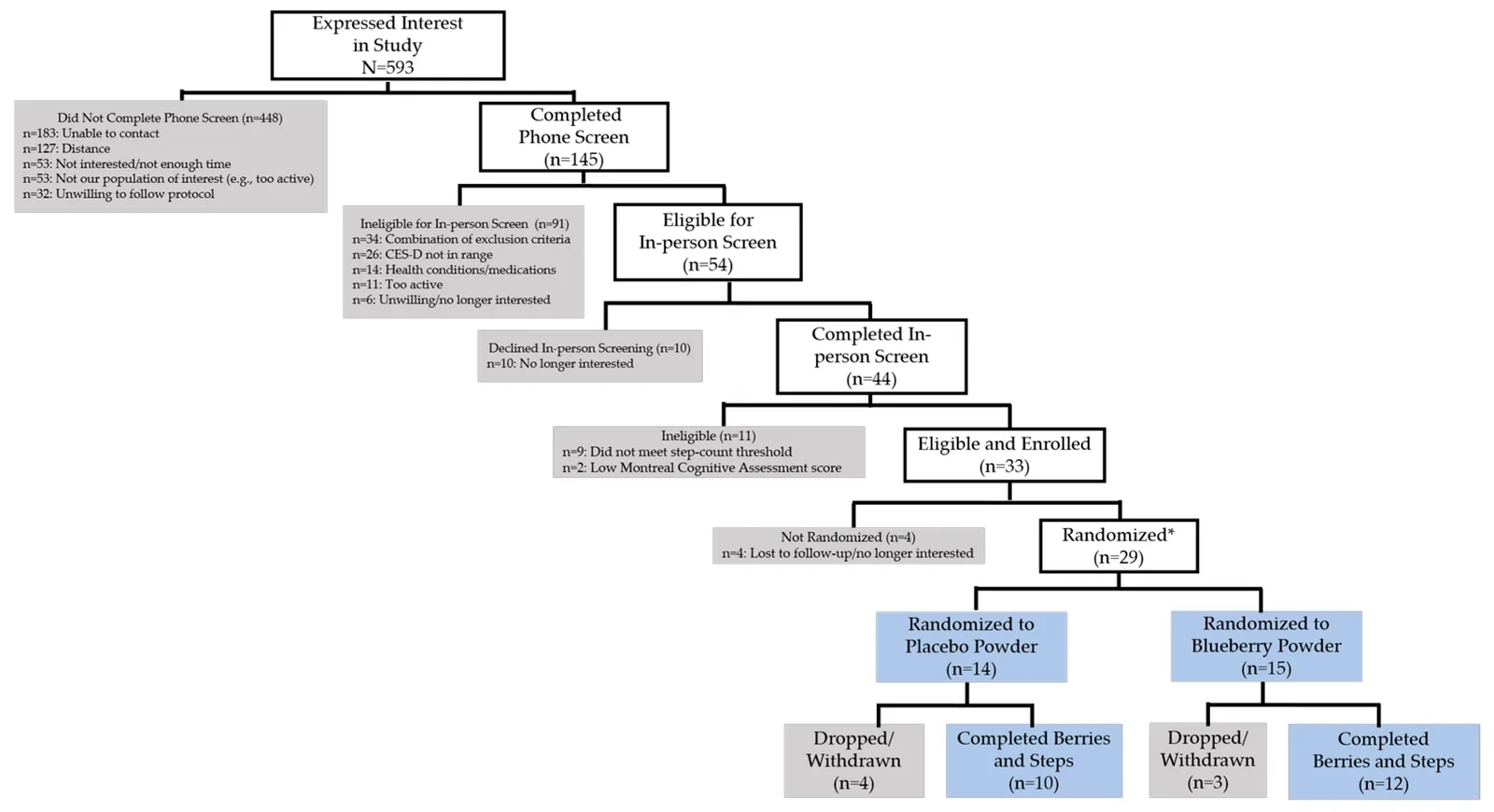
Study flow chart. This figure shows the flow of study participants throughout the recruitment process of the Berries and Steps Study. Reasons for ineligibility or withdrawal are noted in the gray boxes. The blue boxes represent those randomized and/or those that completed the intervention. * Within our randomized sample, there were 2 individuals that were relatively close to the step count threshold. Because it was within a reasonable amount that could be attributed to error of the device that estimated step count, the two participants were included in the study after consultation with the IRB. CES-D, Center for Epidemiological Studies Depression Scale.

A flow chart outlining the process of participant recruitment is shown in Figure 1. Out of 593 individuals that expressed interest in our study, a total of 145 individuals were screened over the telephone for the Berries and Steps Study. Reasons for the 91 individuals that were ineligible included CES-D scores outside our desired range (n = 26), pre-existing health conditions/use of exclusionary medications (n = 14), being too active (n = 11), unwilling/no longer interested (n = 6), or a combination of the former reasons (n = 34). There were 54 individuals eligible for an in-person screen, of which 10 individuals declined participation in the in-person screen. Of the 44 individuals that completed the in-person screen, 11 were ineligible due to low cognitive scores (n = 2) or not meeting our step count threshold (n = 9). This resulted in 33 individuals being eligible and enrolled in our study. However, 4 individuals did not complete their baseline visit. Thus, a total of 29 individuals were randomized to our intervention, which occurred over a total of approximately 14 months (April 2023–June 2024).

### 3.2. Baseline Characteristics of Randomized Participants

The 29 randomized participants had a mean (SD) age of 72 (±5) years and were 79% female (n = 23), primarily white (94%, n = 27) and had relatively high levels of education (Table 1). Of the 29 individuals, 7 individuals dropped from the study. When comparing those who completed the intervention to those who withdrew from the study, those that withdrew were relatively similar to those who completed the intervention. Those that withdrew appear to be slightly younger, have lower point estimates of self-reported motivation, and higher point estimates of step count (Table 1); however, the differences between these groups were not statistically tested.

Of the 29 individuals, 14 were randomized to the placebo, while 15 were randomized to the blueberry group. Groups were relatively well-balanced at baseline (Supplementary Table S1).

### 3.3. Recruitment, Retention, and Drop-Out Rates

Table 2 shows outcomes of study feasibility (e.g., recruitment rate, retention rate) at various stages of the enrollment processes. The pre-specified, primary feasibility outcome was the randomized participant retention rate (e.g., the percentage of participants that were randomized and completed the intervention). We found that 76% (95%CI: 59–93; n = 22 out of 29) of the individuals randomized to the intervention went on to complete the intervention. A total of 7 individuals withdrew from the study (n = 4 placebo, n = 3 blueberry). The reasons for withdrawal included gastrointestinal symptoms (n = 3 placebo; n = 1 blueberry), lack of motivation/inability to commit the time (n = 2 blueberry), and concerns with weight (n = 1 placebo).

### 3.4. Compliance and Method of Powder Consumption

**Figure 2:**
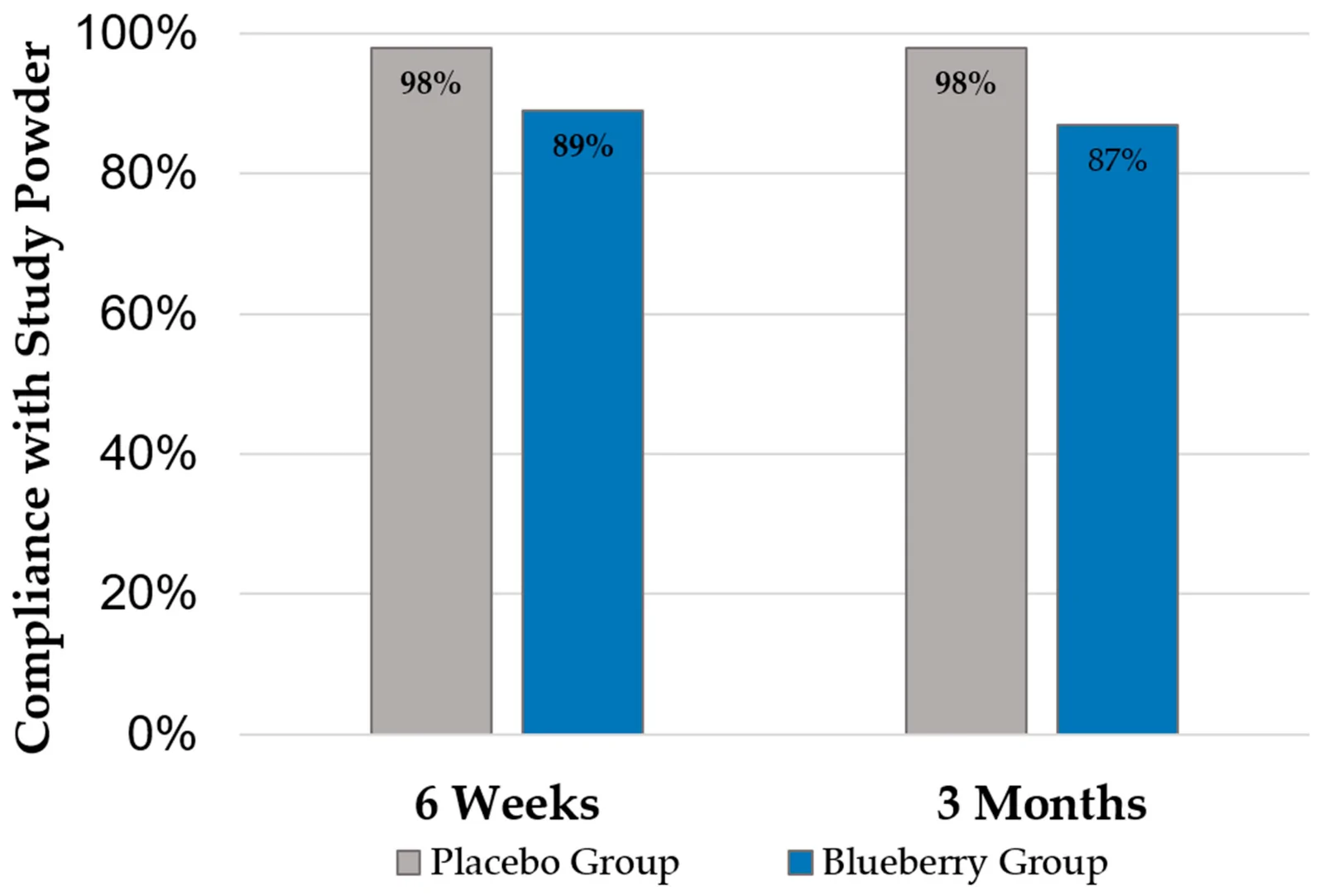
Compliance with study powder among individuals that completed the intervention. The compliance with study powder (defined as percentage of actual doses consumed divided by the intended doses) is presented by group (placebo vs. blueberry) at the mid-point of the intervention (6 weeks) and the final follow-up (3 months). Data from individuals that dropped during the intervention is not included. The gray bars correspond to the placebo group, while the blue bars correspond to the blueberry group.

Participants who completed the study self-reported relatively high compliance. At the 6-week midpoint visit, compliance (i.e., self-reported number of packets consumed out of packets allotted) in the placebo group was 98%, while compliance in the blueberry group was 89%. After 3 months, compliance in the placebo group remained at 98%; however, compliance decreased slightly to 87% in the blueberry group (Figure 2). There were three individuals in the blueberry group who only consumed half of the planned dose mid-way through the intervention, stating it was “too much powder.” One of the three individuals recently had gastric bypass surgery, and the two others did not want to continue if they had to consume the entire 48g dose each day. When including the seven individuals that dropped from the intervention, the compliance rates for the total duration participants were on study dropped to 84% in the placebo group and 77% in the blueberry group.

Of the cited methods for powder consumption, mixing the powder with water was implemented the most frequently in both groups (57% of doses in placebo, and 39% of doses in blueberry; Table 3). Most methods were utilized similarly across groups, except the method of making the powder into a jam or spread, which was reported more commonly in the blueberry group (20% compared to the placebo 3%).

### 3.5. Compliance with Wearable Device

In participants who completed the 12-week study, the compliance rates with the wearable device were 95% in the placebo group and 85% in the blueberry group. Data on compliance with the wearable device for the entire randomized sample was not available.

### 3.6. Safety

In total there were 10 AEs reported from eight participants during our study (Table 4). Thus, two individuals in the placebo group had two AEs each. The reported AEs occurred similarly across groups (n = 6 in 4 individuals in the placebo group, n = 4 in 4 individuals in the blueberry group). No SAEs were reported. Not all AEs resulted in the participant withdrawing from the study. Of the eight individuals that expressed AEs, four withdrew from the study. An additional three withdrew from the study that were not related to AEs (e.g., not enough time commitment).

## 4. Discussion

The goal of this pilot study was to assess recruitment/retention feasibility, adherence, and safety of implementing a daily blueberry intervention in a group of older adults with mild depressive symptoms and low activity. We enrolled 29 individuals who met the inclusion/exclusion criteria, and 22 completed the intervention. Our results provide evidence that implementing a trial that asks older adults to consume 48g of freeze-dried blueberry or placebo powder daily for 3 months may be feasible. While the pre-specified threshold for retention rate that we chose to indicate a feasible intervention was not met (pre-specified threshold: 80%, observed threshold: 76%), other indicators of study feasibility help further support the study’s feasibility. Individuals that completed the intervention, reported relatively high compliance with the study intervention powders (≥87%) over 3 months. Furthermore, despite a relatively large dose of blueberry powder (equivalent to approximately 2 cups of fresh berries per day) there were minimal adverse events and no serious adverse events, which provides additional confidence of the study’s feasibility.

It may be hypothesized that it is difficult to identify and recruit older adults with mild depressive symptoms and low activity for a dietary intervention. Those with mild depressive symptoms may have anhedonia and lack motivation/willingness to engage in a dietary intervention. However, we were able to successfully identify 29 willing and eligible individuals over 14 months of recruitment. This equates to enrolling approximately two individuals per month, which is in line with other estimates from studies performed by our group [29]. Recruitment of this population could potentially be faster, since our recruitment rate was partially limited due to low staffing resources and having a small number of wearable devices available for the study. There was also a considerable drop in the number of individuals that expressed interest to those that completed a telephone screen. We believe this is due to the use of an online recruitment firm that delivers messages to a very large number of individuals. This large drop from expressed interest to completed telephone screen is seen in three other studies that utilized the same recruitment firm, as reported in our previous work [29].

We pre-specified that the study would be considered feasible if the retention rate of those randomized was ≥80%. Our retention rate was 76% (95% CI: 59–93) for the overall group. While the pre-specified threshold of 80% was not met, our observed point estimate for the retention rate of the whole group was close to the target. We want to emphasize that the pre-specified estimate is within the 95% confidence interval and it is possible that the ‘true’ retention rate could be at least 80% (95% CI 59–93). However, with a small sample size, our estimate lacks precision. It is worth noting that the group-specific retention rates were 80% for the blueberry and 71% for the placebo group. This provides further evidence that the blueberry intervention itself may be feasible.

Nevertheless, the study design could benefit from slight modifications. Evaluation of attrition rates may provide insight on how to improve the feasibility of the intervention. Estimates of attrition vary across the literature (5–20%), but are higher (up to 30%) in older adults with chronic conditions [30,31]. Although the attrition rate in our study was relatively high (24%), the rates were similar across groups. One could speculate that this suggests both intervention products were equally tolerable. Those who withdrew tended to have lower motivation scores than those who completed. The lack of motivation is not surprising given our population had mild depressive symptoms. Therefore, in future studies it may be worth over-sampling individuals with lower motivation to account for the attrition and improve retention rates in a larger study.

Furthermore, ~60% of those that dropped from the study cited gastrointestinal issues (n = 3 placebo, n = 1 blueberry). While it is difficult to determine if these gastrointestinal issues were pre-existing, occurring due to the volume of food consumed, or due to powder type (placebo vs. blueberry), it appears that the blueberry powder itself was well-tolerated. The majority of those that cited gastrointestinal issues occurred in the placebo (75%). We speculate that the make-up of the placebo powder drove the dropouts rather than the volume. However, our small study is not able to confirm our hypothesis and other studies that have used similar powders should explore our speculation. The placebo powder was primarily made of maltodextrin, glucose/fructose, a vitamin/mineral mix, and additional flavoring and coloring. It has recently been reported in the literature that maltodextrin may cause gastrointestinal symptoms and modifications [32,33]. Thus, it is important to note that our placebo powder may not be completely inert, especially at this high dose. Future studies could consider alternative placebo constituents and implement a graded dose to help each individual adjust to the amount and ingredients of the powder to potentially avoid gastrointestinal effects. Studies could also consider implementing a run-in period during which the individuals test the powders to pre-emptively identify individuals who may not respond well to the powders. However, that approach could potentially unblind the participant to powder assignment.

Also, with introduction of higher levels of fiber from our proposed dose of the freeze-dried blueberry powder, one might expect an increase in reports of gastrointestinal distress and/or AEs. However, AEs were relatively similar across groups, with more disclosed in the placebo group. Since there were AEs related to gastrointestinal distress in both groups (n = 3 in placebo; n = 3 in blueberry), we cannot confidently attribute these reports to the levels of fiber.

Among individuals that completed the study, compliance with the study powder was relatively high (≥87%) despite a larger dose than most other blueberry studies (most prior studies provided <48g of powder, which is the dose of our current study ~2 cups of fresh blueberries) [34,35,36,37,38,39,40,41,42]. However, it is important to note that there were three individuals in the blueberry group who only consumed 50% of the intended dose for the majority of the intervention. One of the individuals had a previous gastric bypass surgery, which reduced the size of their stomach and limited their ability to consume large quantities of food. Therefore, studies using higher doses may want to consider excluding such individuals and anticipate that there may be lower compliance with the blueberry powder. Despite three individuals only consuming 50% of our dose, participants still maintained high levels of compliance. When evaluating the compliance in the entire randomized sample, compliance estimates dropped to 84% in the placebo group and 77% in the blueberry group. However, these estimates are likely an underestimation since we did not have compliance data from all participants and we conservatively assumed they only took one dose. Therefore, the compliance data in those that completed the intervention could be an overestimation, whereas the compliance data in the randomized sample could be an underestimation.

We allowed individuals to consume the powder in a variety of ways (e.g., mixed with water, made as a shake, sprinkled on cereal). Most participants reported mixing the powder with water. In the blueberry group, a higher percentage of participants mixed the powder into a jam, which may be due to the fiber content in the blueberry powder that provides more structure when made into a jam. Although this study introduced another variable of method of consumption, we hypothesize that this helped maintain high adherence and would encourage future studies to implement similar methods.

Finally, our population of interest was older adults with mild depressive symptoms and low activity. However, there are many ways to quantify low activity (e.g., self-reported hours of sedentary behavior, steps/day, etc.). Furthermore, the thresholds that are used to define individuals with low activity vary across the literature, with most thresholds between 3000 and 5000 steps/day [43,44,45,46]. We used the definition of self-reporting sitting for at least 8 h a day and a step count < 7500 steps per day, which is suggestive of “low activity” [45]. Ultimately, we used a relatively liberal step count threshold to identify individuals with low activity, which may have resulted in misclassification. If we had excluded those on the higher end of the step count, one would assume that the recruitment/retention rates would be lower than those we present here.

During recruitment we observed that many individuals were excluded due to self-reporting they had type two diabetes (T2D). We excluded such individuals out of an abundance of caution. Our proposed dose of blueberry intake would provide an additional 44 g of carbohydrates per day (28 g of sugar, ~11 g of fiber), which could cause blood sugar spikes in those whose T2D is not well managed. Given that T2D often coexists with depressive symptoms and low activity, it is worth exploring the loosening of exclusion criteria to enroll individuals with T2D who are on a stable treatment regimen, especially since there were minimal safety concerns in our study. Inclusion of this group of individuals may improve recruitment rates.

Our results must be contextualized with the limitations of our study. Our sample size was relatively small, as most pilot studies limit the sample size to estimate feasibility outcomes. Furthermore, our cohort is primarily white, well-educated individuals and our results are not generalizable to the larger population. Our feasibility estimates may differ substantially in populations of other educational, socioeconomic, or cultural backgrounds. This was a single-site study, conducted in the Boston area, further limiting generalizability to those living in a metropolitan region. Our study utilized self-reported compliance among individuals that completed the intervention, which may overestimate true adherence. More objective measures should be considered for future studies (e.g., return of used/unused powder packets and/or urinary flavonoid metabolites). Finally, we implemented a relatively high threshold step count to identify individuals with low activity, which positively impacted our recruitment and retention rates. If recruiting for individuals with low activity using a more conservative threshold, additional recruitment challenges may be introduced that are not captured in our work.

## 5. Conclusions

Together these data provide support for the feasibility of implementation of a dietary intervention that provides a relatively high dose of blueberry powder for 3 months in a unique population of older adults—those with low activity and mild depressive symptoms. The observed retention rate was close to our pre-specified threshold, adherence was relatively high among participants that completed the study, and adverse events were minor. When designing similar interventions in the future, investigators should consider over-sampling populations with low motivation, exploring alternative placebos, and implementation of an experimental “run-in” period. Our data on recruitment feasibility, compliance, and safety are instrumental in preparing the next phase of an intervention. More studies reporting these data, particularly in other educational, socioeconomic, cultural backgrounds, would benefit the larger scientific community.

## Figures and Tables

**Table 1:** Characteristics of berries and steps participants at baseline.

	All Participants	All Completed Participants	All Withdrawn Participants
	(n = 29)	(n = 22)	(n = 7)
	Mean ± SD or n (%)		
Age (y)	72 ± 5	73 ± 5	70 ± 4
Sex (female)	23 (79)	17 (77)	6 (86)
Height (meters)	1.6 ± 0.1	1.6 ± 0.1	1.6 ± 0.1
Weight (kg)	81 ± 18	79 ± 19	86 ± 17
Systolic Blood Pressure (mmHg)	128 ± 12 *	128 ± 14 *	129 ± 7
Diastolic Blood Pressure (mmHg)	74 ± 7 *	75 ± 7 *	72 ± 6
Self-Identified Ethnicity/Race			
White	27 (94)	20 (90)	7 (100)
Asian	1 (3)	1 (5)	0 (0)
Jewish	1 (3)	1 (5)	0 (0)
Highest Level of Education			
Some College/Vocational	2 (7)	2 (9)	0 (0)
Associate’s Degree	2 (7)	2 (9)	0 (0)
Bachelor’s Degree	3 (10)	2 (9)	1 (14)
Higher Education (Master’s, Doctoral, etc.)	22 (76)	16 (73)	6 (86)
History of Smoking	15 (52)	12 (55)	3 (43)
MoCA Score (range of instrument: 0–30 points)	27.3 ± 1.8	27.3 ± 1.8	27.29 ± 2.1
CES-D Revised Score (inclusion criteria: 4–16 points)	9.3 ± 4.1	9.5 ± 4.2	8.7 ± 3.7
Anti-Depressant Use	6 (21)	4 (18)	2 (29)
MPAM-R Score (range of instrument 30- 210 points)	151 ± 22	155 ± 21	138 ± 23
MSPSS Score (range of instrument 0–7 points)	6 ± 1	6 ± 1	6 ± 1
Avg of Median Step Count Before Intervention **	6103 ± 1282	5955 ± 1314	6566 ± 1137

* Excludes an individual who went on anti-hypertensives after baseline. ** Represents the mean (SD) value of the median daily step count that was captured prior to baseline. MoCA, Montreal Cognitive Assessment. CES-D Revised, Center for Epidemiological Studies-Depression Scale Revised. MPAM-R, Motives for Physical Activities Measure—Revised. MSPSS, Multidimensional Scale of Perceived Social Support.

**Table 2:** Feasibility outcomes of the berries and steps study.

Outcome	Brief Description	Rate or n
Telephone Screen Recruitment Rate (# of participants enrolled in study / # of telephone screened participants) × 100	The percentage of volunteers telephone screened that were enrolled in the study.	(33 / 145) × 100 = 22.8% 22.8% of people who completed the telephone screen were enrolled in the study.
In-person Screen Recruitment Rate (# of participants enrolled in study / # of in-person screened participants) × 100	The percentage of volunteers screened that were enrolled in the study.	(33 / 44) × 100 = 75% 75% of people who completed the screening enrolled in the study.
Telephone Screen Retention Rate (# of participants completed study / # of telephone screened participants) × 100	The percentage of volunteers telephone screened that ultimately completed the intervention.	(22 / 145) × 100 = 15.2% 15.2% of people who completed the telephone screen went on to complete the intervention.
In-person Screen Retention Rate (# of participants completed study / # of in-person screened participants) × 100	The percentage of screened volunteers who ultimately completed the intervention.	(22 / 44) × 100 = 50% 50% of people who completed the screening went on to complete the intervention.
Randomized Participant Retention Rate (# of participants completed study / # of participants randomized) × 100	The percentage of participants that were randomized who ultimately completed the intervention.	(22 / 29) × 100 = 76% 76% of people that were randomized went on to complete the intervention.

#: number.

**Table 3:** Methods of powder consumption across groups.

	Completed Participants (n = 22)	Placebo (n = 10)	Blueberry (n = 12)
Method	n (%)		
Water	833.5 (47)	458.5 (57)	375 (39)
Shake	216 (12)	111 (14)	105 (11)
Almond Milk	181.5 (10)	66.5 (8)	115 (12)
Jam	219 (12)	22.5 (3)	196.5 (20)
Cereal	105.5 (6)	69 (8)	36.5 (4)
Other Beverage	102 (6)	82 (10)	20 (2)
Yogurt/Cottage Cheese	33.5 (2)	0 (0)	33.5 (4)
Other Method	79 (5)	1 (0)	78 (8)
Total N	**1770 (100)**	**810.5 (100)**	**959.5 (100)**

**Table 4:** Description of adverse events collected during study.

AE Number	Participant	Group	Brief Description of Adverse Event	Approx Time on Study Powder	Relationship to Study Participation	Withdrawal from Study (Y/N)
1	A	Placebo	Vomiting for 1 day	6 weeks	Possible	No
2	A	Placebo	Pre-existing condition of high blood pressure	Throughout the study	Unlikely	No
3	B	Placebo	Increased frequency of loose stools, loose stools	4 weeks	Possible	Yes
4	C	Placebo	Irritated esophagus	<1 week	Probable	Yes
5	D	Placebo	Bloating	<1 week	Possible	Yes
6	D	Placebo	Unpleasant taste of study powder that “lingered” throughout the day.	<1 week	Possible	Yes
7	E	Blueberry	Muscle pain from pre-existing condition	8 weeks	Unlikely	No
8	F	Blueberry	Increase in frequency of bowel movements	<1 week	Possible	Yes
9	G	Blueberry	Bloating	4 weeks	Possible	No
10	H	Blueberry	Constipation	6 weeks	Possible	No

## Data Availability

The dataset supporting the conclusions of this article is available upon request to the corresponding author under conditions of appropriate ethical oversight, upon investigator approval and execution of a data use agreement. Materials will include the de-identified data presented in this manuscript, data dictionary, and analytic plan.

## Supplementary Materials

The following supporting information can be downloaded at: https://www.mdpi.com/article/10.3390/nu18213694/s1, Table S1. Baseline Characteristics of Randomized Berries and Steps Participants By Group.
